# A novel scale-up strategy for cultivation of BHK-21 cells based on similar hydrodynamic environments in the bioreactors

**DOI:** 10.1186/s40643-021-00393-3

**Published:** 2021-08-13

**Authors:** Xiaonuo Teng, Chao Li, Xiaoping Yi, Yingping Zhuang

**Affiliations:** State Key Laboratory of Bioreactor Engineering, East China University of Science and Technology, Shanghai, 200237 China

**Keywords:** BHK-21 cells, Bioreactor, Computational fluid dynamics, Hydrodynamic characteristics, Scale-up

## Abstract

The scale-up of animal cell cultivation is important but remains complex and challenging. In the present study, we propose a novel scale-up strategy for baby hamster Syrian kidney-21 (BHK-21) cell cultivation based on similar hydrodynamic environments. The hydrodynamic characteristics of the different scale bioreactors were determined by computational fluid dynamics (CFD) and further correlated with the agitation speed. The optimal hydrodynamic environment for cell cultivation and vaccine production was determined from the cultivation experiments of BHK-21 cells in 5-L laboratory-scale bioreactors equipped with different impellers at various agitation speeds. BHK-21 cell cultivation was scaled up from 5-L to 42-, 350-, and 1000-L bioreactors by adjusting the agitation speed to make the hydrodynamic features similar to those in the 5-L bioreactor, especially for the shear rate in the impeller zone (*γ*_imp_) and energy dissipation rate in the tank bulk zone (*ε*_tan_). The maximum cell density and cell aggregation rate in these scaled-up bioreactors were in the range of 4.6 × 10^6^ ~ 4.8 × 10^6^ cells/mL and 16 ~ 20%, which are comparable to or even better than those observed in the 5-L bioreactor (maximum cell density 4.8 × 10^6^ cells/mL, cell aggregation rate 21%). The maximum virus titer of 10^8.0^ LD_50_/mL achieved in the 1000-L bioreactor was close to 10^8.3^ LD_50_/mL that obtained in the 5-L bioreactor. Hence, the scale-up strategy proposed in this study is feasible and can efficiently facilitate the scale-up processes of animal cell cultivation.

**Supplementary Information:**

The online version contains supplementary material available at 10.1186/s40643-021-00393-3.

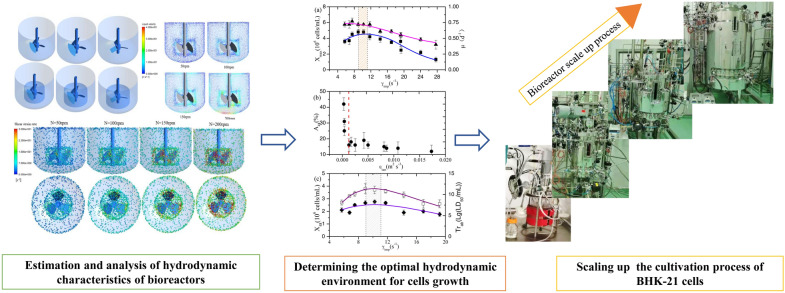

## Introduction

Hydrophobia is a zoonotic infectious disease caused by the rabies virus with the highest mortality rate in the world (Brunker & Mollentze 2018; Martinez 2000). Vaccination is efficient, safe, and the most effective way to prevent rabies. At present, cell strains including baby hamster syrian kidney-21 (BHK-21), HDC, Vero, and chicken embryo cells can produce the rabies vaccine (Cai et al. 2015; Kallel et al. 2002; Moro et al. 2019; Trabelsi et al. 2006). BHK-21 cells are an ideal host for producing rabies vaccine, since they can be easily cultured and yield high levels of virus (Kallel et al. 2002). The suspension culture of BHK-21 cells in large-scale bioreactors has been used to produce certain vaccines. For example, production lines with bioreactors from 1000 to 4000 L have been established to produce foot-and-mouth disease vaccine in China (Li et al. 2019). However, rabies vaccine production using BHK-21 cells has mainly been conducted in roller bottles or small-scale bioreactors, which is a low-productivity and labor-intensive processes. Today, it is still a great challenge to scale up the cultivation process of BHK cells for producing the rabies vaccine.

Computational fluid dynamics (CFD) has been widely used for decades in the biochemical engineering fields. It is used to obtain the detailed hydrodynamic characteristics of bioreactors, and mainly provides a basis for optimizing and scaling up the cultivation process of microorganisms (Cappello et al. 2021; Haringa et al. 2018; Xia et al. 2008). Recently, CFD has also been applied in the characterization, optimization, and scale-up or scale-down of bioreactors for animal cell cultivation (Borys et al. 2019, 2018; Li et al. 2020; Menshutina et al. 2020; Villiger et al. 2018). Borys et al. (2018) evaluated seven commonly used scale-up parameters including volume average velocity, volume average shear stress rate, volume average energy dissipation, Reynolds number, impeller tip speed, power input, and maximum shear rate to scale up embryonic stem cell cultivation. They observed the corresponding agitation rates associated with different scale-up parameters differed greatly, and found that maintaining the volume average energy dissipation rate was the best method for scaling up. Li et al. (2019) reported that the equivalent volumetric power (*P*/*V*) was an appropriate scale-down strategy of foot-and-mouth vaccine production by suspension BHK-21 cells. However, these traditional scale-up or scale-down approaches mainly emphasize the consistency of one characteristic parameter. Consequently, it is difficult to ensure that the most of the hydrodynamic characteristics are similar in the different bioreactors during scale-up, which introduces significant variation into the hydrodynamic environment and further affects cell growth (Borys et al. 2018). Furthermore, we have proposed, in a previous study, a scale-up method for shear-sensitive animal cells based on the similarity of the shear environment inside bioreactors. In this way, cultivation of *Spodoptera frugiperda* (Sf9) cells was successfully scaled up from the lab-scale to industrial-scale bioreactor (Li et al. 2020). However, while the shear environments were kept similar during the scale-up processes, the other hydrodynamic parameters varied greatly, which led to the unstable performance of cells productivity. Thus, in the present study, we propose another scale-up strategy that focuses on the similarity of hydrodynamic environments rather than the shear environment. Compared to the other scale-up method previous reported, the most significant feature of the approach is to keep the hydrodynamic environment as similar as possible between the lab-scale and large-scale bioreactor instead of only considering one scale-up factor. First, CFD was used to quantitatively characterize the hydrodynamic characteristics of the different scale bioreactors. Then, the optimal hydrodynamic environment was determined by culture experiments in the 5-L bioreactors. Finally, the scale-up experiments of BHK-21 cell culture were carried out in the 42-, 350-, and 1000-L bioreactor to verify the scale-up strategy.

## Materials and methods

### Description of the bioreactors

**Fig. 1:**
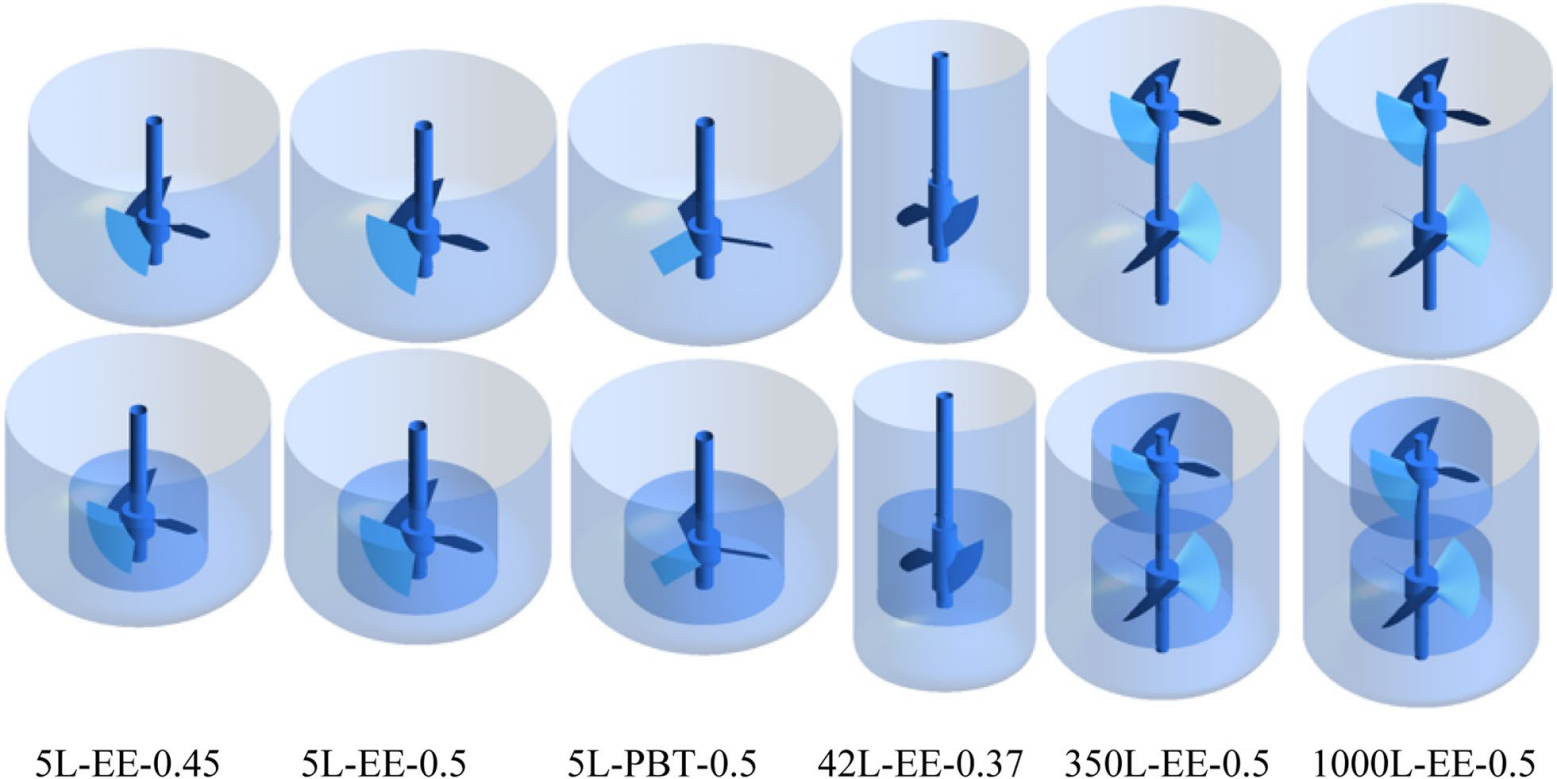
Schematic diagram of the three-dimensional bioreactors and impeller zones for CFD simulation (the text at the bottom indicates the type of each bioreactor, namely, “bioreactor volume-impeller type-ratio of impeller diameter to tank diameter”)

Different bioreactors equipped with various impellers were used to investigate the scale-up of BHK-21 cells (Fig. 1 and Table 1). Three 5-L bioreactors, which were equipped with impellers of different types and diameters, were employed to determine the optimal hydrodynamic characteristics of BHK-21 cell cultivation and rabies vaccine production. For the scale-up of cell cultivation, 42-, 350-, and 1000-L bioreactors were used. The details of the bioreactors used are shown in Table 1. The 5-L bioreactor is equipped with a three-blade ‘Elephant Ears’ impeller (EE) or three-pitched-blade turbine impeller (PBT). The EE impellers were used in the 42-, 350-, and 1000-L bioreactors.

**Table 1:** Bioreactors used in this study for BHK-21 cell cultivation

Bioreactor type^a^	5L-EE-0.45	5L-EE-0.5	5L-PBT-0.5	42L-EE-0.37	350L-EE-0.5	1000L-EE-0.5
Tank volume (L)	5	5	5	42	350	1000
Tank diameter (mm)	192	192	192	290	644	914
Impeller diameter (mm)	86.4	96.0	96.0	108.0	323.5	429.7
Impeller diameter/tank diameter	0.45	0.50	0.50	0.37	0.50	0.50
Culture level height (mm)	158.0	158.0	158.0	492.5	737.6	1067.6
Load volume (L)	3.50	3.50	3.50	31.0	238.0	682.0
Impeller type	EE	EE	PBT	EE	EE	EE
Number of impellers	Single	Single	Single	Single	Double	Double

^a^Bioreactor type is expressed as “bioreactor volume-impeller type-ratio of impeller diameter to tank diameter”

### CFD simulation setup

The unstructured tetrahedral mesh was generated for bioreactors using ANSYS ICEM CFD15.0 (ANSYS Inc., USA). The grids in the impeller zone were refined to accurately capture the hydrodynamic characteristics, as the mixing in this zone was extremely strong. The grid independent test was performed in advance to obtain an accurate simulation result. Thereby, 5.9 × 10^5^, 1.1 × 10^6^, 1.6 × 10^6^, and 2.1 × 10^6^ final appropriate computational elements were created for the 5-, 42-, 350-, and 1000-L reactors, respectively.

The multiple reference frame (MRF) method was applied to simulate the impeller rotation. The mixture two-phase model with standard $k - \varepsilon$ model for turbulence was used to simulate gas–liquid flow behavior, because the gas and liquid both exist with a large Reynolds number (> 10,000) in the bioreactor. The working liquid within the bioreactor was assumed to be water, because the physicochemical properties of BHK-21 culture are close to water.

The mass conservation and momentum equation for each phase *i* is given:

1 $$\frac{\partial }{\partial t}(\alpha_{i} \rho_{i} ) + \mathop{\nabla }\limits^{\rightharpoonup} \cdot (\alpha_{i} \rho_{i} {\mathbf{V}}_{i} ) = 0$$

2 $$\frac{\partial }{\partial t}(\alpha_{i} \rho_{i} {\mathbf{V}}_{i} ) + \mathop{\nabla }\limits^{\rightharpoonup} \cdot (\alpha_{i} \rho_{i} {\mathbf{V}}_{i} {\mathbf{V}}_{i} ) = - \alpha_{i} \mathop{\nabla }\limits^{\rightharpoonup} P + \alpha_{i} \rho_{i} g + \mathop{\nabla }\limits^{\rightharpoonup} \cdot \left( {\alpha_{i} \overline{R}_{i} } \right) + {\mathbf{F}}_{{\mathbf{i}}} ,$$

where *ρ*_*i*_ is density of phase *i*, **V**_i_ is the phase velocity, *g* is the gravitational acceleration, and *P* is the mean pressure shared by both phases. $\overline{R}_{i}$ is the combined viscous and Reynolds (turbulent) stress for phase *i*, and **F**_i_ is the interphase momentum transfer term, which is the sum of all the interfacial forces acting on phase i from the other phase.

For the simulation, the gas sparger was simplified as a point sparger for gas injection with a flow rate of 0.05 vvm at the bottom of the bioreactor. In addition, the gas phase in the reactor was simulated as a mono-disperse gas to simplify the solution of the flow field (Kazemzadeh et al. 2018). The drag model of Schiller and Naumann was used. The outlet was set as the degassing boundary, which allows gas in the dispersed phase to escape only from the top surface and not from the continuous phase. A second-order spatial discretization scheme was used for the momentum, pressure, turbulent kinetic energy, and dissipation rate, and the SIMPLE scheme was used for pressure–velocity coupling (Kazemzadeh et al. 2018). The simulations were performed by ANSYS CFX 15.0 (ANSYS Inc., USA). A computer equipped with 8 cores (2.4 GHz Intel® Core™ i5 CPU and 16 RAM) was used to run the simulations. The convergence was achieved when the residuals for the solved quantities reached at least 10^–4^ and the volume average gas holdup within the bioreactor was not changed. The required computational time for each simulation was 40–70 h depending on grid numbers. The CFD model has been validated via comparison of the simulated velocities with the results obtained by the particle image velocimetry (PIV) experiment in our previous work (Li et al. 2020), ensuring the simulation result with a high accuracy.

### Calculation of hydrodynamic characteristic parameters

The different hydrodynamics characteristic parameters were calculated to represent the hydrodynamic environment in the bioreactor. The volume average liquid velocity (*U*), energy dissipation rate (*ε*), and shear rate (*γ*) were directly obtained using post-processing software ANSYS CFX-POST 15.0. EDCF is an energy dissipation/circulation function, which more reasonably characterizes the shear level experienced by the cells. The calculation formula for EDCF is as follows (Rocha-Valadez et al. 2007):

3 $${\text{EDCF}} = \frac{P}{{kD^{3} }} \cdot \frac{1}{{t_{{\text{c}}} }}$$

4 $$k = \frac{\pi }{4} \cdot \frac{W}{D}$$

5 $$t_{{\text{c}}} = \frac{{V_{{\text{L}}} }}{{Q_{{\text{f}}} }},$$

where *P* is the stirring power (w), *D* is the impeller diameter (m), *W* is the height of the stirring blade (m), *t*_c_ is the circulation time (s), *V*_L_ is the load volume in the bioreactor (m^3^), and *Q*_f_ is the discharge flow of impeller (m^3^ s^−1^).

The stirring power of the bioreactor is calculated based on the agitation speed and torque and the formula is as follows (Rocha-Valadez et al. 2007):

6 $$P = 2\pi NM,$$

where *N* is the agitation speed (rps), and *M* is the torque (N m).

The EE and PBT impeller mainly generate axial flow. Therefore, the formula for calculating the discharge flow is as follows (Tang et al. 2015):

7 $$Q_{{\text{f}}} { = }2\pi \int\limits_{0}^{D/2} {xv_{{{\text{axial}}}} } \left( x \right){\text{d}}x,$$

where *D* is the impeller diameter and *v*_axial_ is the component of the flow velocity in the axial direction (m s^−1^).

Additionally, the tip velocity of impeller (*U*_tip_) is calculated by $U_{{{\text{tip}}}} = \pi DN$, where *D* is the impeller diameter (m), and *N* is the agitation speed (rps) (Hardy et al. 2017).

The gas–liquid mass transfer coefficient (*K*_L_*a*) indicates the oxygen supply capacity in the bioreactor. The *K*_L_*a* of the bioreactor is calculated as follows (Ndiaye et al. 2018):

8 $$K_{{\text{L}}} a = K_{{\text{L}}} \cdot a$$

9 $$K_{{\text{L}}} = \frac{2}{\sqrt \pi }\sqrt {\frac{{D_{{O_{2} }} \left| {U_{{\text{g}}} - U_{{\text{l}}} } \right|}}{{d_{{{\text{mean}}}} }}}$$

10 $$a = \frac{{6\alpha_{g} }}{{d_{mean} }},$$

where *D*_O2_ is the molecular diffusion coefficient of oxygen in water, and *U*_g_ and *U*_l_ represent the gas and liquid velocity (m s^−1^), respectively. *d*_mean_ is the bubble diameter (m) and $\alpha_{{\text{g}}}$ is the gas phase fraction.

### Experiments of BHK-21 cell cultivation in the bioreactors

BHK-21 cells were used as the cell line for rabies vaccine production. Serum-free BS-SFM media, which was obtained from Suzhou Womei Biotechnology Co., Ltd in China, were applied for BHK-21 cell cultivation.

The most direct and convenient way to change the flow field environment in the bioreactor is to adjust the agitation speeds and the type of impeller. Different hydrodynamic environments were created in the bioreactors. For this purpose, we used four different agitation speeds (50, 100, 150, and 200 rpm) to culture BHK-21 cells in the 5-L bioreactors equipped with different impeller types (EE and PBT) and sizes (diameter ratio of impeller to tank 0.45 and 0.5).

Seed cells were prepared in the flasks. Cells were cultured at 37 ± 0.1 °C in a CO_2_ incubator. Then, seed cells were incubated into 5-L bioreactors. The initial cell density of 5-L bioreactor was 0.8 × 10^6^ cells/mL. The temperature and pH were maintained at 37 ± 0.1 °C and 7.1 ± 0.1, respectively. During the culture process, the concentration of dissolved oxygen was controlled in the range of 40–50%, while it was regulated at a level of 20–30% at the stage of rabies virus reproduction by adjusting input amount of pure oxygen into bioreactors. Samples were analyzed every 24 h for cell density and percentage of agglomerated cells. Three parallel tests were carried out in the 5-L bioreactors to determine the optimal hydrodynamic environments for BHK-21 cells cultivation.

BHK-21 cell cultivation process was scaled up from 5-L to 42-, 350-, and 1000-L bioreactors. Seed cells were prepared in the shaking flasks and 5-L bioreactor. The initial inoculum density was 0.8 × 10^6^ cells/mL. The temperature and pH were controlled at 37 ± 0.1 °C and 7.1 ± 0.1. The agitation speed of the large-scale bioreactor (42-, 350-, and 1000-L) was calculated according to the mathematical relationship between the hydrodynamic characteristic parameters and agitation speed, as well as the optimal hydrodynamic characteristics determined from the 5-L bioreactor. Accordingly, the agitation speeds for the 42-, 350-, and 1000-L bioreactors were chosen as 80, 65, and 50 rpm, respectively, which ensured that the hydrodynamic characteristics of the bioreactors were most similar to that in the 5-L bioreactor. For these batch cultures of BHK-21cells in the 42-, 350-, and 1000-L bioreactors, rabies virus was not inoculated in the cells culture. However, another batch culture was conducted with rabies virus injection and amplification in a 1000-L bioreactor to observe the rabies virus production. In the rabies virus amplification experiment, all culture conditions were controlled to be the same with previous experiment of cells cultivation. However, rabies virus was inoculated into cell cultures at 48 h with MOI = 1.0 during cultivation process. In addition, the temperature was subsequently regulated from 37 ± 0.1 °C to 34 ± 0.1 °C. Dissolved oxygen concertation was adjusted to ~ 25%. Samples were taken daily to measure the cell morphology, cell density, and virus titer.

A Counterstar cell counter (ALIT Life Science, China) was used to determine the viable cell density and cell agglomeration ratio. For cell density counting, culture sample was first mixed with trypan blue dye solution (1:1, v/v), then added to counting chamber, and moved into an automated cell counter. Cell number and aggregation rate were directly measured by the cell counter. In addition, the viable cells can be dyed blue by trypan blue dye solution, and detected by the instrument. Cell viability (%) was calculated by

11 $${\text{Viability}}\;(\% ) = \frac{{X_{{\text{t}}} - X_{0} }}{{X_{{\text{t}}} }} \times 100\% ,$$

where *X*_t_ is the total number of cells (cells/mL) and *X*_0_ is the number of the death cells (cells/mL).

For determination of rabies virus titer, the samples stored at − 80 ℃ were repeatedly freeze thaws for three times, centrifuged at 1000 rpm for 15 min, and then, the supernatant was gradually diluted by 10 times using phosphate-buffer saline (PBS, 0.04 mol/L, pH = 7.6) with five dilutions of 10^–3^ ~ 10^–7^. Finally, 0.03 mL dilution was injected into the brains of 10 mice with the weight of 13 ~ 16 g. The mice were fed for 14 days; those that died within 96 h after injection were excluded from the statistical analysis. The survival number of mice on day 5 and the number of mice that died on days 5–14 were recorded to calculate the virulence of the virus (LD_50_). The significant differences of culture results in the 5-L bioreactors were assessed by using ANOVA with statistically difference at a level of 1%.

## Results and discussion

### Modeling and analysis of hydrodynamic characteristics of the bioreactors

**Fig. 2:**
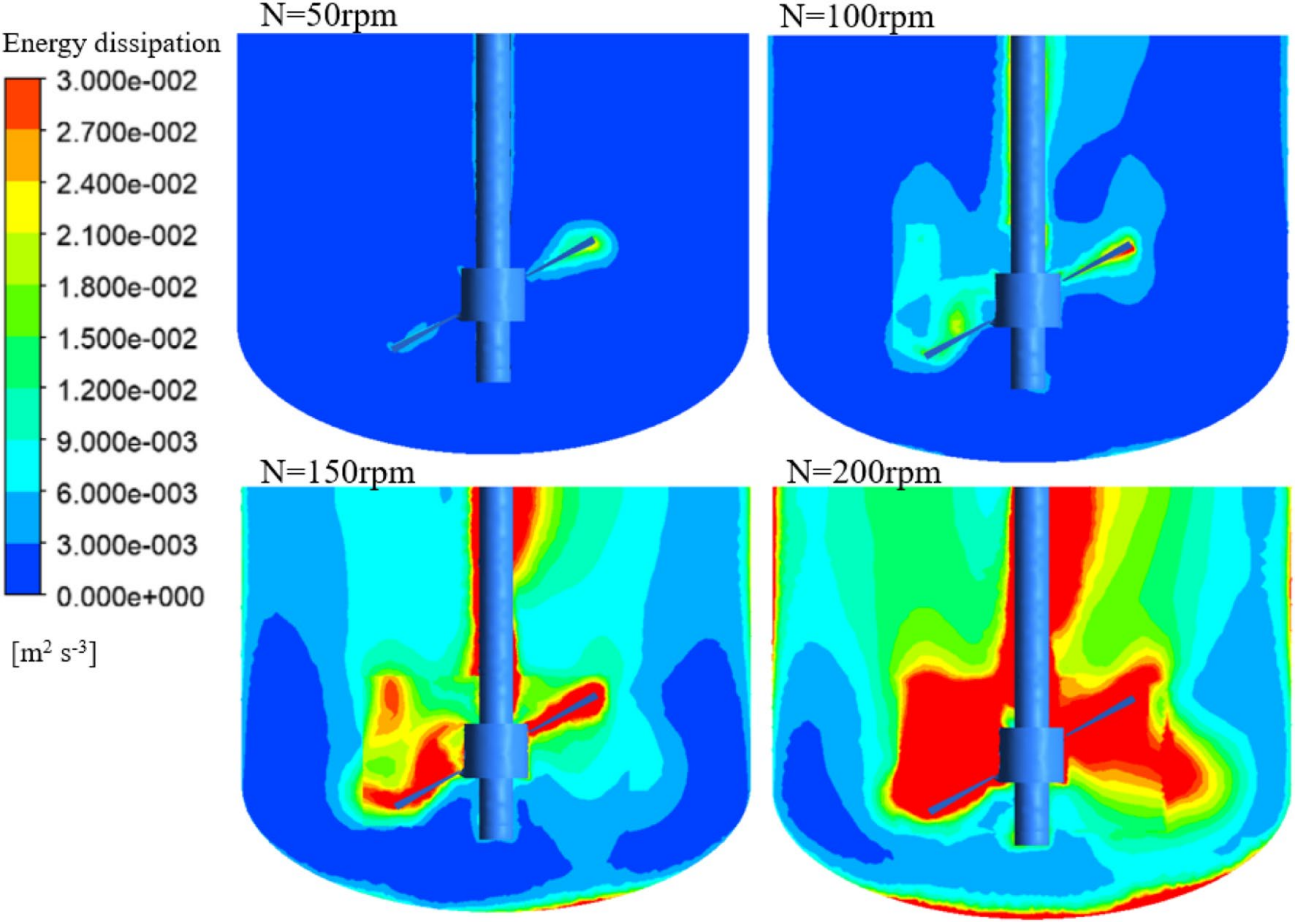
Distribution of energy dissipation of 5 L bioreactor equipped with EE impeller (impeller diameter/tank diameter 0.45) at different agitation speeds

**Fig. 3:**
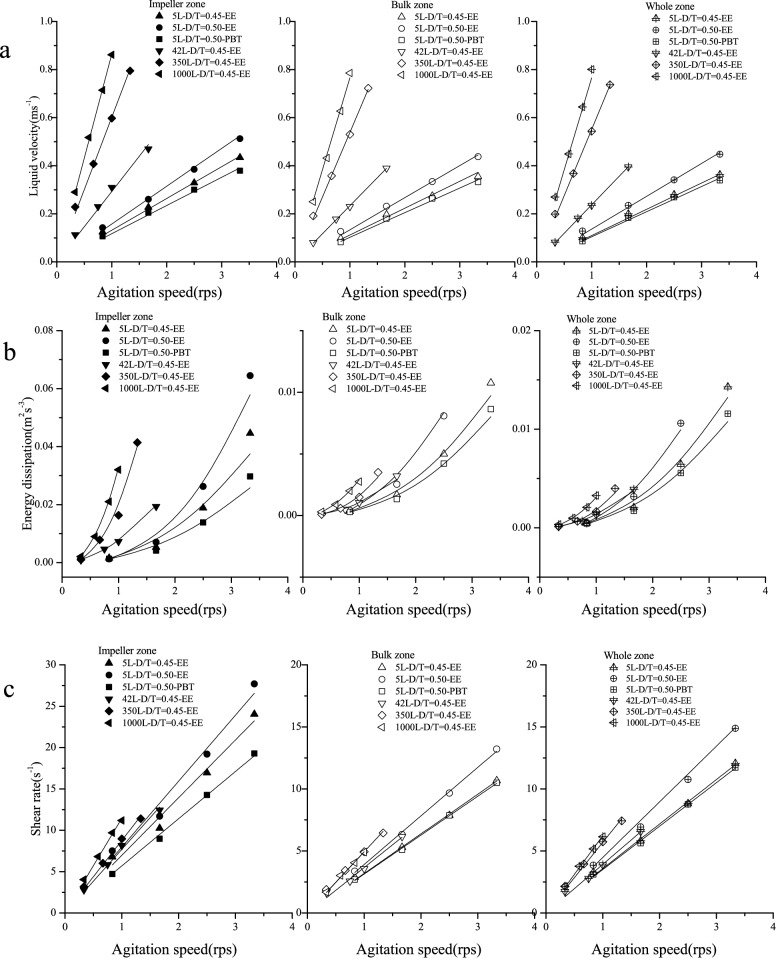
The volume average liquid velocity (**a**), energy dissipation (**b**), and shear rate (**c**) in the impeller zone, bulk zone, and entire tank under different agitation speeds

The hydrodynamic characteristics of bioreactors significantly affect the growth of animal cells (Kazemzadeh et al. 2018). CFD simulation showed that the flow field within the bioreactors was not uniform (Additional file 1: Fig. S1). The liquid velocity in the impeller zone was higher than that in the tank bulk zone. Additionally, the volume average liquid velocity gradually increased as the agitation speed increased (Fig. 2). The volume average liquid velocity of the 1000-L bioreactor was the highest and subsequently decreased in the 350-L, 42-L, and 5-L bioreactors at the same agitation speed. A mathematical fitting was performed to find the relationship between the volume average liquid velocity and agitation speed. The volume average liquid velocities in the impeller zone (*U*_imp_) and bulk zone (*U*_tan_), as well as the average liquid velocity of the entire bioreactor (*U*_ave_) fit well with the agitation speed (Additional file 1: Table S1). In addition, energy dissipation is an important hydrodynamic parameter commonly associated with the shear environment in the bioreactor for cell cultivation (Rocha-Valadez et al. 2007). A gradient of the energy dissipation rate in the bioreactor also exists. Clearly, the energy dissipation rate was highest near the stirring blade (Fig. 2). The volume average energy dissipation rates in the different bioreactors are shown in Fig. 3. The volume average energy dissipation rate increased significantly as the agitation speed increased. The largest bioreactor had the highest energy dissipation rate (1000 L > 350 L > 42 L > 5 L) at the same agitation speed. In addition, the impeller with the larger diameter produces a higher energy dissipation rate, since more power is generated and delivered to the bioreactor (compare bioreactor 5 L-EE-0.45 with bioreactor 5 L-EE-0.5). Nonlinear fitting of the energy dissipation rate with the agitation speed indicated that the energy dissipation rate has a power function relationship with the agitation speed (Fig. 3 and Additional file 1: Table S2). The shear rate is a parameter widely used to quantify the shear force in the bioreactor. It has a great impact on animal cell growth (Li et al. 2020). CFD simulation showed that the shear rate was also distributed unevenly in the bioreactor (Additional file 1: Fig. S2). Moreover, the volume average shear rate linearly increased with the agitation speed (Fig. 3 and Additional file 1: Table S3). This relationship between shear rate and agitation speed has been reported in other studies (Borys et al. 2018; Campesi et al. 2009; Li et al. 2020).

**Fig. 4:**
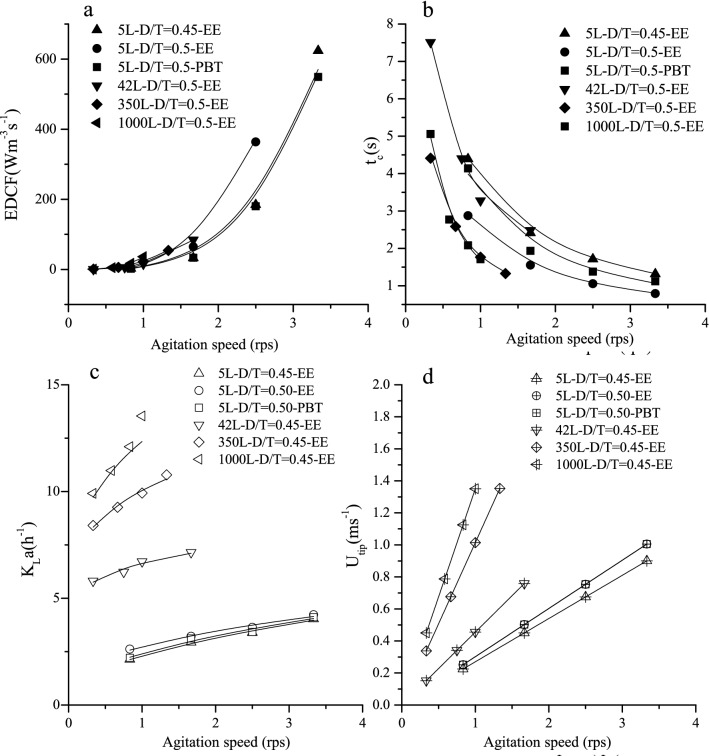
The EDCF (**a**), *t*_c_ (**b**), *K*_L_*a* (**c**), and *U*_tip_ in bioreactors under different agitation speeds

EDCF is also considered to be closely related to the shear environment and has been used as a scale-up criterion (Hardy et al. 2017; Li et al. 2020). EDCF in the bioreactor increased non-linearly with the increase of the agitation speed (Fig. 4). However, the circulation time (*t*_c_) decreased as the agitation speed increased. The discharge flow of the impeller increased with the increasing agitation speed, which led to a shorter circulation time. The numerical fitting results show that EDCF has a power function relationship with the agitation speed, while the circulation time has a negative power function relationship with the agitation speed (Additional file 1: Table S4). *K*_L_*a* represents the oxygen supply capacity in the bioreactor and is extensively used as a scale-up parameter (Villiger et al. 2018). The mathematical fitting shows that *K*_L_*a* has a power function relationship with the agitation speed (Fig. 4 and Additional file 1: Table S5). In addition, the tip velocity of the impeller is linearly related to the agitation speed and the impeller diameter.

### Determining the optimal hydrodynamic environment for cell growth

**Fig. 5:**
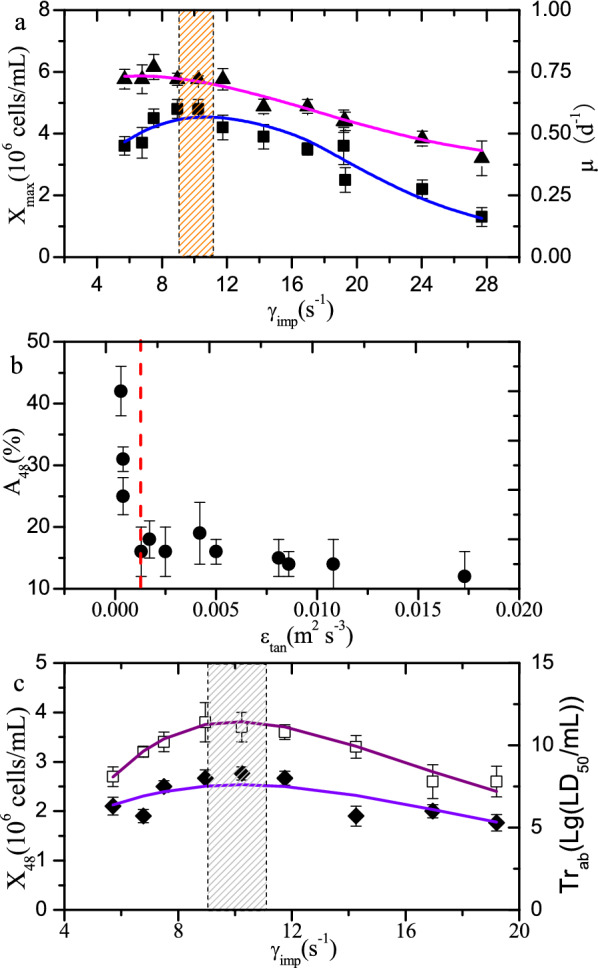
Effects of shear environment in the 5-L bioreactor on BHK-21 cell growth performance and virus titer. Effect of shear rate in the impeller zone (*γ*_imp_) on the highest viable cell density (*X*_max_, filled square) and average specific growth rate of BHK-21 cells during the first 24 h post-inoculation (*μ*_24_, filled triangle)_;_ effect of *ε*_tan_ on the ratio of aggregated cells at 48 h (*A*_48_, filled circle); and effect of γ_imp_ on the viable cell density at 48 h (*X*_48_, Square) and rabies virus titer (*T*_rab_, filled diamond)

The hydrodynamic environment in the bioreactor, which includes mixing, shear, and mass transfer, significantly affects the growth of animal cells (Borys et al. 2018). Therefore, determining the optimal hydrodynamic environment in the bioreactor is a prerequisite for the scale-up of the culture process for a given cell line. Thus, the batch culture experiments of BHK-21 cells were carried out in three 5-L bioreactors at different agitation speeds (Additional file 1: Fig. S3). The analysis of variance (ANOVA) indicates that there is a statically significant difference between the culture results obtained from the bioreactors equipped with the different types and sizes of impellers, showing hydrodynamic environment significantly influences cell growth and aggregation rate (*P* < 0.01, *F* = 12.2 for maximum cell density, *F* = 33.3 for cell aggregation rate). It has been reported that the average shear rate especially at the impeller zone has a great impact on the performance of cell growth (Kazemzadeh et al. 2018; Li et al. 2020). Thus, the effect of the volume average shear rate in the impeller zone (*γ*_imp_) on the cell-specific growth rate during first 24 h after inoculation (*μ*_24_) and the maximum cell density (*X*_max_) were analyzed. The maximum level of cell density and *μ*_24_ were achieved when *γ*_imp_ is in the range 8.5–11.0 s^−1^ (Fig. 5). *X*_max_ and *μ*_24_ decreased greatly when *γ*_imp_ continued to increase. One problem in the serum-free culture of BHK-21 cells is cell aggregation. The low agitation speed generates a low shear force, which favors improved cell viability. However, the low mixing intensity is not sufficient to counteract the tendency of cell aggregation, which leads to an increase in the rate of cell agglomeration that does not favor viral growth (Borys et al. 2019). The energy dissipation rate in the tank zone (*ε*_tan_) is always used to represent the ability of disrupting cell agglomeration (Borys et al. 2018, 2019). As shown in Fig. 5, the ratio of aggregated cells at 48 h (*A*_48_) significantly increased when the *ε*_tan_ value was less than 1.3 × 10^–3^ m^2^ s^−3^. However, the improvement of cell agglomeration was not significant when *ε*_tan_ was greater than 1.3 × 10^–3^ m^2^ s^−3^. Thus, the condition *ε*_tan_ ≥ 1.3 × 10^–3^ m^2^ s^−3^ should be maintained in the bioreactor for the efficient cultivation of BHK-21 cells. It should be noted that the parameters of shear rate and energy dissipation rate are not independent, and they are related to each other (Villiger et al. 2018). Energy dissipation rate is used as a characteristic parameter to evaluate the shear effect on cell aggregation, because it has a positive correlation with the hydrodynamic shear rate. In the present study, cell density and aggregation rate are, respectively, associated with *γ*_imp_ and *ε*_tan_, since it has been reported that cell growth rate and aggregation are significantly sensitive to the shear rate and energy dissipation rate, respectively (Borys et al. 2018; Li et al. 2019).

In the batch cultures, the maximum cell density was reached at 72 h during the culture process (Additional file 1: Fig. S3). The specific cell growth rate in the time range of 48–72 h was significantly lower than that in the 24–48 h range. Accordingly, the rabies virus was inoculated at a multiplicity of infection (MOI) of 1.0 at 48 h in the BHK-21 cell culture process. It was observed that agitation speed of 200 rpm has a significant adverse impact on the growth of BHK-21 cells. Therefore, viral production experiments were only conducted with agitation speeds of 50 rpm, 100 rpm, and 150 rpm. The maximum viral titer positively correlated with the cell density (Fig. 5). The optimal range of *γ*_imp_ for viral titer production was also in the range 8.5–11 s^−1^, which is consistent with that required to achieve the maximum cell density.

The range of agitation speeds that meet the two key characteristic conditions, namely $8.5 \le \gamma_{{{\text{imp}}}} \le 11.0$ s^−1^ and $\varepsilon_{\tan } \ge 0.0013$ m^2^ s^−3^, were calculated for three 5-L bioreactors based on the mathematical fitting model above-mentioned (Table 2). The intersection range of the agitation speeds derived from the optimal *γ*_imp_ and *ε*_tan_ was determined. Furthermore, the ranges of the corresponding hydrodynamic characteristic parameters were calculated according to the intersection range of the agitation speed. In this way, the characteristic profile of the optimal hydrodynamic environment was obtained for efficient cell culture and virus production (Table 3).

**Table 2:** Ranges of agitation speeds for BHK-21 cells cultured in the different 5-L bioreactors as required by the optimal key hydrodynamic parameters

Bioreactor type	Key flow field parameters	Agitation speed (rpm)	Intersection of agitation speed (rpm)
5L-EE-0.45	$8.5{0} \le \gamma_{{{\text{imp}}}} \le 11.0$(s^−1^)	_73.2 ≤ *N* ≤ 94.7_	_88.4 ≤ *N* ≤ 94.7_
	$\varepsilon_{\tan } \ge 0.0013$(m^2^ s^−3^)	_*N* ≥ 88.4_	
5L-EE-0.5	$8.5{0} \le \gamma_{{{\text{imp}}}} \le 11.0$(s^−1^)	_64.0 ≤ *N* ≤ 82.8_	_75.4 ≤ N ≤ 82.8_
	$\varepsilon_{\tan } \ge 0.0013$(m^2^ s^−3^)	_*N* ≥ 75.4_	
5L-PBT-0.5	$8.5{0} \le \gamma_{{{\text{imp}}}} \le 11.0$(s^−1^)	_89.4 ≤ *N* ≤ 115.7_	_96.3 ≤ *N* ≤ 115.7_
	$\varepsilon_{\tan } \ge 0.0013$(m^2^ s^−3^)	_*N* ≥ 96.3_

**Table 3:** Optimal hydrodynamic characteristic values of 5-L bioreactors at the optimized agitation speed ranges

Bioreactor type	5L-EE-0.45		5L-EE-0.5		5L-PBT-0.5		Min	Max
Agitation speed (rpm)	88.4	94.7	75.4	82.8	96.3	115.7		
*γ*_imp_ (s^−1^)	10.25	11.01	10.04	11.00	9.18	11.01	9.18	11.01
*γ*_ave_ (s^−1^)	5.27	5.67	5.65	6.19	5.64	6.76	5.27	6.76
*ε*_tan_ (m^2^ s^−3^)	0.0013	0.0015	0.0013	0.0017	0.0013	0.0021	0.0013	0.0021
*ε*_ave_ (m^2^ s^−3^)	0.0018	0.0021	0.0015	0.0019	0.0019	0.0029	0.0015	0.0029
EDCF (w m^3^ s^−1^)	23.58	31.20	20.42	30.01	29.48	61.32	20.42	61.32
*t*_c_ (s)	2.69	2.53	1.98	1.82	2.13	1.79	1.79	2.69
*K*_L_*a* (h^−1^)	2.75	2.84	2.97	3.06	2.97	3.21	2.75	3.21
*U*_tip_ (m s^−1^)	0.40	0.43	0.38	0.42	0.49	0.58	0.38	0.58
*U*_ave_ (m s^−1^)	0.16	0.17	0.17	0.19	0.17	0.20	0.16	0.20

### Determination of appropriate agitation speeds for scaled-up bioreactors

The hydrodynamic characteristics in the 42-, 350-, and 1000-L bioreactors were required to be controlled as similar as those in the 5-L bioreactor to provide an appropriate environment for cell growth. The corresponding agitation speeds for the 42-, 350-, and 1000-L bioreactors were estimated according to the mathematical fitting model and *ψ*_BHK-21_ (Table 4). The suitable agitation speeds in the scaled-up bioreactors were reasonably chosen to make most of the hydrodynamic characteristics to be in the range of *ψ*_BHK-21_. *γ*_imp_ and *ε*_tan_ were considered in priority to other parameters. Hence, the agitation speeds of the 42-, 350-, and 1000-L bioreactors for cell culture were finally determined as 80, 65, and 50 rpm, respectively (Table 5). The corresponding hydrodynamic characteristics in these scaled-up bioreactors were further predicted based on the fitting equations. The most characteristic parameters in each scaled-up bioreactor are in the range of *ψ*_BHK-21_. However, *K*_L_*a*, *U*_tip_, and *U*_ave_ in these bioreactors are larger than the maximum level of *ψ*_BHK-21_.

**Table 4:** Agitation speeds calculated for the optimal hydrodynamic parameters of BHK-21 cells cultivated in 42-, 350-, and 1000-L bioreactors

Hydrodynamic parameter	Optimal range of hydrodynamic parameters		Agitation speed (rpm)					
			42 L		350 L		1000 L	
	Min	Max	Min	Max	Min	Max	Min	Max
*γ*_imp_ (s^−1^)	9.18	11.01	71.7	85.9	62.7	75.2	48.1	57.6
*ε*_tan_ (m^2^ s^−3^)	0.0013	0.0021	68.0	85.4	56.7	68.6	41.1	51.5
*γ*_ave_ (s^−1^)	5.27	6.76	80.5	103.3	55.5	71.2	50.8	65.1
*ε*_ave_ (m^2^ s^−3^)	0.0015	0.0029	64.3	88.4	57.1	74.0	42.6	58.1
EDCF (w m^−3^ s^−1^)	20.42	61.32	65.5	91.0	59.0	82.8	51.8	69.8
*t*_c_ (s)	1.79	2.69	85.6	55.8	102.6	57.4	57.1	37.6
*K*_L_*a* (h^−1^)	2.75	3.21	0.07	0.24	0.03	0.08	0.06	0.12
*U*_tip_ (m s^−1^)	0.38	0.58	67.2	102.6	22.4	34.3	16.9	25.8
*U*_ave_ (m s^−1^)	0.16	0.20	40.4	50.5	17.4	21.8	12.2	15.2

**Table 5:** The hydrodynamic parameters of large-scale bioreactors at the agitation speed calculated as the desirable hydrodynamic environment for the culture of BHK-21 cells

Parameter	Optimal range of hydrodynamic parameters		Hydrodynamic parameters of scaled-up bioreactors		
	Min	Max	42 L (80 rpm)	350 L (65 rpm)	1000 L (50 rpm)
*γ*_imp_ (s^−1^)	9.18	11.01	10.38	9.51	9.73
*ε*_tan_ (m^2^ s^−3^)	0.0013	0.0021	0.0019	0.0018	0.0021
*γ*_ave_ (s^−1^)	5.27	6.76	5.30	6.17	5.29
*ε*_ave_ (m^2^ s^−3^)	0.0015	0.0029	0.0024	0.0208	0.0022
EDCF (w m^−3^ s^−1^)	20.42	61.32	41.52	27.98	19.35
*t*_c_ (s)	1.79	2.69	2.03	2.43	2.00
*K*_L_*a* (h^−1^)	2.75	3.21	6.92	10.21	11.91
*U*_tip_ (m s^−1^)	0.38	0.58	0.46	1.10	1.15
*U*_ave_ (m s^−1^)	0.16	0.20	0.32	0.60	0.66

### Scale-up of BHK-21 cell cultivation in the bioreactors

**Fig. 6:**
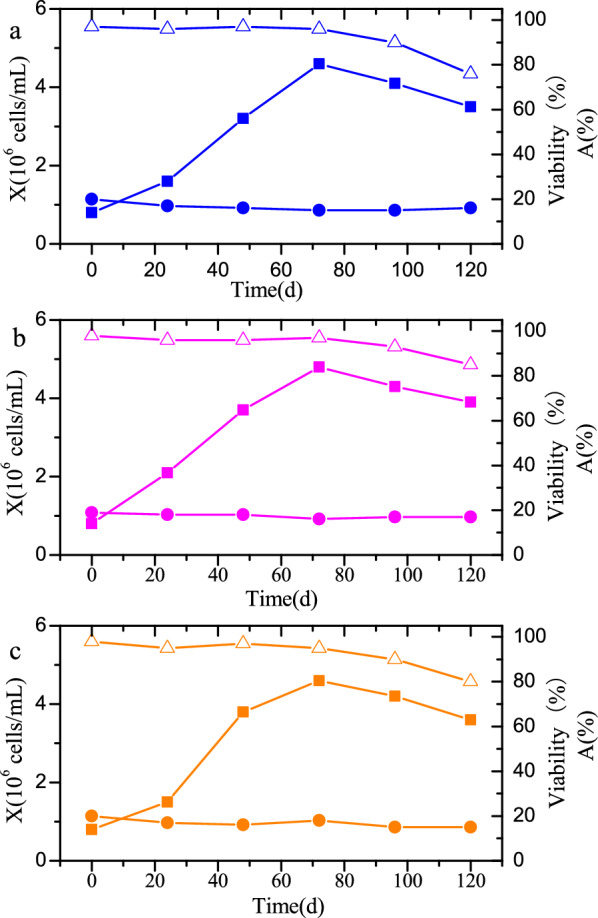
Viable cell density (*X*, filled square), viability (triangle), and the ratio of aggregated cells (*A*, filled circle) in batch cultures of BHK-21 cells in 42- (**a**), 350- (**b**), and 1000-L (**c**) bioreactors

In the 42-L bioreactor, BHK-21 cells were cultured at an agitation speed of 80 rpm, which almost generated a similar hydrodynamic environment to that of the 5-L bioreactor. The cell growth profile is shown in Fig. 6. The BHK-21 cell density reached 4.6 × 10^6^ cells/mL at 72 h. Meanwhile, the cell agglomeration rate was maintained at about 17% during the entire culture process. The cell culture result in the 42-L bioreactor is comparable to the optimal cell cultivation achieved in the 5-L bioreactor (maximum cell density 4.8 × 10^6^ cells/mL; cell agglomeration rate 21%). Furthermore, BHK-21 cells were cultured in a 350-L bioreactor with an agitation speed of 65 rpm. The maximum cell density also reached 4.8 × 10^6^ cells/mL with a cell agglomeration rate of ~ 18% during the entire process. This result is even better than the best performance of cells cultured in the 5-L bioreactors. Finally, BHK-21 cell culture was scaled up to the 1000-L bioreactor with an agitation speed of 50 rpm. A maximum cell density of 4.6 × 10^6^ cells/mL and an average cell agglomeration rate of 17% were obtained. These results are comparable to the results in the 5-, 42-, and 350-L bioreactors, which indicates that it is feasible to scale-up the BHK-21 cell culture from a lab-scale to an industrial-scale by creating similar hydrodynamic environments.

In the cultivation of BHK-21 cells, cell agglomeration should be considered (Borys et al. 2018) alongside the cell growth rate. Cell agglomeration is affected by many factors such as media components, cell age, bioreactor type, and culture conditions (Moreira et al. 1995). Cell agglomeration can prevent nutrients and oxygen from entering the inside of the cell cluster, which may lead to metabolic disorders in the culture organisms, morphological changes, and even cell death. The damage to cell and viral structures by shear forces is also avoided (Heath and Kiss 2007). However, cell agglomeration will also affect the infection efficiency of the virus and reduce virus productivity. Thus, controlling the cell agglomeration ratio is of great significance to improve the virus productivity (Borys et al. 2018). A critical energy dissipation rate of 1.3 × 10^–3^ m^2^ s^−3^ was obtained in the 5-L bioreactor. The cell agglomeration rate during the scale-up process was mainly kept in a normal range of 16–20%, which indicates that a good cell status is maintained in the scaled-up bioreactors.

**Fig. 7:**
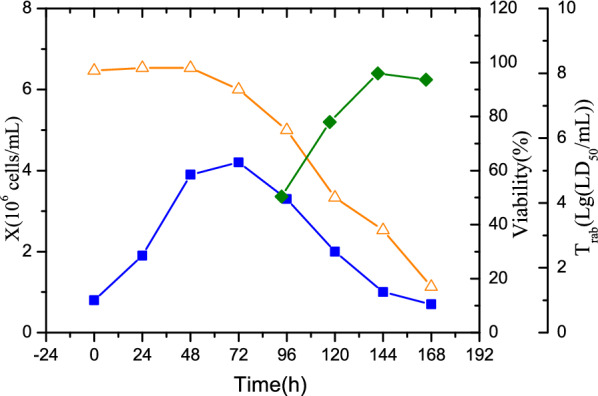
Viable cell density (*X*, filled square), viability (triangle), and virus titer (*T*_rab_, filled diamond) in the batch culture of BHK-21 cells in the 1000-L bioreactor

In another batch culture, the infection and multiplication of the rabies virus were also examined in the 1000-L bioreactor. The rabies virus was inoculated into BHK-21 cells culture at 48 h. The cell growth, viability, and virus titer are shown in Fig. 7. After inoculation of virus, cell growth is obviously inhibited with a significant reduced cell viability rate. At 144 h, a maximum virus titer of 10^8.0^ LD_50_/mL was achieved, which was close to the best performance in the 5-L bioreactor (10^8.3^ LD_50_/mL). This indicates that rabies vaccine production in an industrial bioreactor was scaled up successfully from the lab-scale bioreactor. Using this approach, we can directly scale up cell growth efficiently from the lab-scale to the industrial-scale bioreactor without extensive testing of agitation rates.

The traditional scale-up of bioprocesses often depends on a single engineering parameter, such as mass transfer coefficient, specific power consumption, mixing time, shear rate, energy dissipation, and impeller tip speed (Garcia-Ochoa and Gomez 2009; He et al. 2019; Möller et al. 2020; Tescione et al. 2015). Thus, it is difficult to make most of the hydrodynamic features in the large-scale bioreactor as similar to those presented in the lab-scale bioreactor. This leads to a decrease of the cell growth and constrains cells and bioproducts’ productivity. Moreover, traditional cell culture scale-up is usually a trial-and-error approach. It is often costly and time-consuming with a high risk of failure (He et al. 2019). In addition, only a few studies on the scale-up of animal cell cultivation have been reported and more efforts should be made in this direction (Borys et al. 2018). In the present study, we proposed a novel scale-up strategy that ensuring the similarity of the hydrodynamic environments in the different scale bioreactors for animal cell culture. The optimal cell growth and virus production in a 5-L bioreactor was successfully replicated in 42-, 350-, and 1000-L bioreactors using this method.

The cell culture performances in the scaled-up bioreactors were almost comparable to those in the 5-L bioreactor even when *K*_L_*a*, *U*_tip_, and *U*_ave_ were not in the optimal range. This indicates that inconsistency in these parameters during the scale-up process has no obvious side effects. In fact, it is difficult to make the flow field in the industrial bioreactor totally identical to that in the lab-scale bioreactor (Möller et al. 2020). However, successful scale-up can still be achieved if most of the primary characteristic parameters of hydrodynamics in the large-scale bioreactor are consistent with those in the lab-scale bioreactors. In addition, a similar hydrodynamic environment in the scaled-up bioreactors can be achieved by adjusting bioreactor structure, impeller type, and impeller size in addition to the regulation of agitation speed. Further research into the hydrodynamic environment of the bioreactor may yield additional benefits for efficient optimization and scale-up of animal cell cultivation.

## Conclusions

The scale-up of animal cell culture is difficult and complex. In the present study, a scale-up strategy based on similar hydrodynamic environments was proposed and further validated by cell culture experiments of BHK-21 cells. The performance of BHK-21 cell cultivation in the 42-, 350-, and 1000-L bioreactors was comparable or even better than that in the lab-scale bioreactor when the hydrodynamic characteristics in the scaled-up bioreactors were controlled to be as similar as possible to those in the 5-L bioreactor. The scale-up method aided by CFD is feasible and effective and has the advantages of saving time and cost. Thus, it is expected that this scale-up strategy will advance the industrial-scale cultivation of BHK-21 and other animal cells.

### Supplementary Information

**Additional file 1: Fig. S1.** Flow field of 5 L bioreactor equipped with EE impeller (impeller diameter/tank diameter 0.45) at different agitation speeds. **Fig. S2** Distribution of shear rate in 5 L bioreactor equipped with EE impeller (impeller diameter/tank diameter 0.45) at different agitation speeds. **Fig. S3** Viable cell density (*X*), viability and aggregated cells ratio (A) of BHK-21 cells at different agitation speeds (■: 50 rpm, ●: 100 rpm, ♦: 150 rpm, ▲: 200 rpm) in 5L-EE-0.45 bioreactors (a), 5L-EE-0.5 bioreactors(b), and 5L-PBT-0.5 bioreactors(c). **Table S1** Fitting models of liquid velocity with agitation speed for different bioreactors. **Fig. S2** Fitting models of energy dissipation with agitation speed for different bioreactors. **Fig. S3** Fitting models of shear rate with agitation speed for different bioreactors. **Fig. S4** Fitting models of *EDCF* and circulation time with rotation speed for different bioreactors. **Fig. S5** Fitting models of *K*_*L*_*a* with agitation speed for different bioreactors.

## Data Availability

Data will be made available upon reasonable request. The cell line is intellectual property of Laboratories and cannot be shared.

## Abbreviations

*A*_48_: Ratio of aggregated cells at 48 h
EE: ‘Elephant Ears’ impeller
PBT: Pitched-blade turbine impeller
*U*_imp_: Liquid velocity in impeller zone (m s^−^^1^)
*U*_tan_: Liquid velocity in the tank bulk zone (m s^−^^1^)
*U*_ave_: Average liquid velocity in the entire zone of bioreactor (m s^−^^1^)
*U*_tip_: Tip velocity of impeller (m s^−^^1^)
*K*_L_*a*: Volumetric mass transfer rate (h^−^^1^)
*EDCF*: Energy dissipation/circulation function (w m^3^ s^−^^1^)
*D*: Impeller diameter (m)
*P*: Stirring power (w)
*t*_c_: Circulation time (s)
*W*: Height of the stirring blade (m)
*V*_L_: Load volume (m^3^)
*Q*_f_: Discharge flow of impeller (m^3^ s^−^^1^)
*N*: Agitation speed (rpm or rps)
*M*: Torque (N m)
*X*_max_: Maximum cell density (cells/mL)
*γ*_imp_: Shear rate in impeller zone (s^−^^1^)
*γ*_tan_: Shear rate in the tank bulk zone (s^−^^1^)
*γ*_ave_: Average shear rate in the entire zone of bioreactor (s^−^^1^)
*ε*_imp_: Energy dissipation rate in impeller zone (m^2^ s^−^^3^)
*ε*_tan_: Energy dissipation rate in the tank bulk zone (m^2^ s^−^^3^)
*ε*_ave_: Average energy dissipation rate in the entire zone of bioreactor (m^2^ s^−^^3^)
*μ*_24_: Specific growth rate of cells during first 24 h after inoculation (h^−^^1^)

## References

[CR1] Borys BS, Roberts EL, Le A, Kallos MS (2018). Scale-up of embryonic stem cell aggregate stirred suspension bioreactor culture enabled by computational fluid dynamics modeling. Biochem Eng J.

[CR2] Borys BS, Le A, Roberts EL, Dang T, Rohani L, Hsu CY-M, Wyma AA, Rancourt DE, Gates ID, Kallos MS (2019). Using computational fluid dynamics (CFD) modeling to understand murine embryonic stem cell aggregate size and pluripotency distributions in stirred suspension bioreactors. J Biotechnol.

[CR3] Brunker K, Mollentze N (2018). Rabies virus. Trends Microbiol.

[CR4] Cai Y, Zhou R, Li S, Huang Q, Yang G, Feng X, Hou W (2015). Study on the quality of lyophilized human diploid cell rabies vaccine using microcarrier technology. J Appl Virol.

[CR5] Campesi A, Cerri MO, Hokka CO, Badino AC (2009). Determination of the average shear rate in a stirred and aerated tank bioreactor. Bioprocess Biosyst Eng.

[CR6] Cappello V, Plais C, Vial C, Augier F (2021). Scale-up of aerated bioreactors: CFD validation and application to the enzyme production by *Trichoderma reesei*. Chem Eng Sci.

[CR7] Garcia-Ochoa F, Gomez E (2009). Bioreactor scale-up and oxygen transfer rate in microbial processes: an overview. Biotechnol Adv.

[CR8] Hardy N, Augier F, Nienow AW, Béal C, Ben Chaabane F (2017). Scale-up agitation criteria for *Trichoderma reesei* fermentation. Chem Eng Sci.

[CR9] Haringa C, Mudde RF, Noorman HJ (2018). From industrial fermentor to CFD-guided downscaling: what have we learned?. Biochem Eng J.

[CR10] He C, Ye P, Wang H, Liu X, Li F (2019). A systematic mass-transfer modeling approach for mammalian cell culture bioreactor scale-up. Biochem Eng J.

[CR11] Heath C, Kiss R (2007). Cell culture process development: advances in process engineering. Biotechnol Prog.

[CR12] Kallel H, Jouini A, Majoul S, Rourou S (2002). Evaluation of various serum and animal protein free media for the production of a veterinary rabies vaccine in BHK-21 cells. J Biotechnol.

[CR13] Kazemzadeh A, Elias C, Tamer M, Ein-Mozaffari F (2018). Hydrodynamic performance of a single-use aerated stirred bioreactor in animal cell culture: applications of tomography, dynamic gas disengagement (DGD), and CFD. Bioprocess Biosyst Eng.

[CR14] Li X-R, Yang Y-K, Wang R-B, An F-L, Zhang Y-D, Nie J-Q, Ahamada H, Liu X-X, Liu C-L, Deng Y, Bai Z-H, Li Y, Liu X-R (2019). A scale-down model of 4000-L cell culture process for inactivated foot-and-mouth disease vaccine production. Vaccine.

[CR15] Li C, Teng X, Peng H, Yi X, Zhuang Y, Zhang S, Xia J (2020). Novel scale-up strategy based on three-dimensional shear space for animal cell culture. Chem Eng Sci.

[CR16] Martinez L (2000). Global infectious disease surveillance. Int J Infect Dis.

[CR17] Menshutina NV, Guseva EV, Safarov RR, Boudrant J (2020). Modelling of hollow fiber membrane bioreactor for mammalian cell cultivation using computational hydrodynamics. Bioprocess Biosyst Eng.

[CR18] Möller J, Hernández Rodríguez T, Müller J, Arndt L, Kuchemüller KB, Frahm B, Eibl R, Eibl D, Pörtner R (2020). Model uncertainty-based evaluation of process strategies during scale-up of biopharmaceutical processes. Comput Chem Eng.

[CR19] Moreira J, Cruz PE, Santana PC, Aunins JG, Carrondo MJT (1995). Formation and disruption of animal cell aggregates in stirred vessels: mechanisms and kinetic studies. Chem Eng Sci.

[CR20] Moro PL, Lewis P, Cano M (2019). Adverse events following purified chick embryo cell rabies vaccine in the vaccine adverse event reporting system (VAERS) in the United States, 2006–2016. Travel Med Infect Di.

[CR21] Ndiaye M, Gadoin E, Gentric C (2018). CO_2_ gas–liquid mass transfer and k_L_a estimation: numerical investigation in the context of airlift photobioreactor scale-up. Chem Eng Res Des.

[CR22] Rocha-Valadez JA, Galindo E, Serrano-Carreón L (2007). The influence of circulation frequency on fungal morphology: a case study considering Kolmogorov microscale in constant specific energy dissipation rate cultures of *Trichoderma harzianum*. J Biotechnol.

[CR23] Tang W, Pan A, Lu H, Xia J, Zhuang Y, Zhang S, Chu J, Noorman H (2015). Improvement of glucoamylase production using axial impellers with low power consumption and homogeneous mass transfer. Biochem Eng J.

[CR24] Tescione L, Lambropoulos J, Paranandi MR, Makagiansar H, Ryll T (2015). Application of bioreactor design principles and multivariate analysis for development of cell culture scale down models. Biotechnol Bioeng.

[CR25] Trabelsi K, Rourou S, Loukil H, Majoul S, Kallel H (2006). Optimization of virus yield as a strategy to improve rabies vaccine production by Vero cells in a bioreactor. J Biotechnol.

[CR26] Villiger TK, Neunstoecklin B, Karst DJ, Lucas E, Stettler M, Broly H, Morbidelli M, Soos M (2018). Experimental and CFD physical characterization of animal cell bioreactors: from micro- to production scale. Biochem Eng J.

[CR27] Xia J-Y, Wang S-J, Zhang S-L, Zhong J-J (2008). Computational investigation of fluid dynamics in a recently developed centrifugal impeller bioreactor. Biochem Eng J.

