# Supplementary material for: A novel scale-up strategy for cultivation of BHK-21 cells based on similar hydrodynamic environments in the bioreactors
Source: Bioresour Bioprocess. 2021 Aug 13;8(1):74. doi: 10.1186/s40643-021-00393-3 (PMC10991166; doi:10.1186/s40643-021-00393-3)
Supplement: Supplementary file 1 — Additional file 1: Fig. S1. Flow field of 5 L bioreactor equipped with EE impeller (impeller diameter/tank diameter 0.45) at different agitation speeds. Fig. S2 Distribution of shear rate in 5 L bioreactor equipped with EE impeller (impeller diameter/tank diameter 0.45) at different agitation speeds. Fig. S3 Viable cell density (X), viability and aggregated cells ratio (A) of BHK-21 cells at different agitation speeds (■: 50 rpm, ●: 100 rpm, ♦: 150 rpm, ▲: 200 rpm) in 5L-EE-0.45 bioreactors (a), 5L-EE-0.5 bioreactors(b), and 5L-PBT-0.5 bioreactors(c). Table S1 Fitting models of liquid velocity with agitation speed for different bioreactors. Fig. S2 Fitting models of energy dissipation with agitation speed for different bioreactors. Fig. S3 Fitting models of shear rate with agitation speed for different bioreactors. Fig. S4 Fitting models of EDCF and circulation time with rotation speed for different bioreactors. Fig. S5 Fitting models of KLa with agitation speed for different bioreactors. [file 40643_2021_393_MOESM1_ESM.docx]

**[Supplementary Material](https://www.springer.com/journal/449/submission-guidelines#Instructions%20for%20Authors_Electronic%20Supplementary%20Material)**

**Fig. S1** Flow field of 5 L bioreactor equipped with EE impeller (impeller diameter/tank diameter 0.45) at different agitation speeds.

**Fig. S2** Distribution of shear rate in 5 L bioreactor equipped with EE impeller (impeller diameter/tank diameter 0.45) at different agitation speeds.


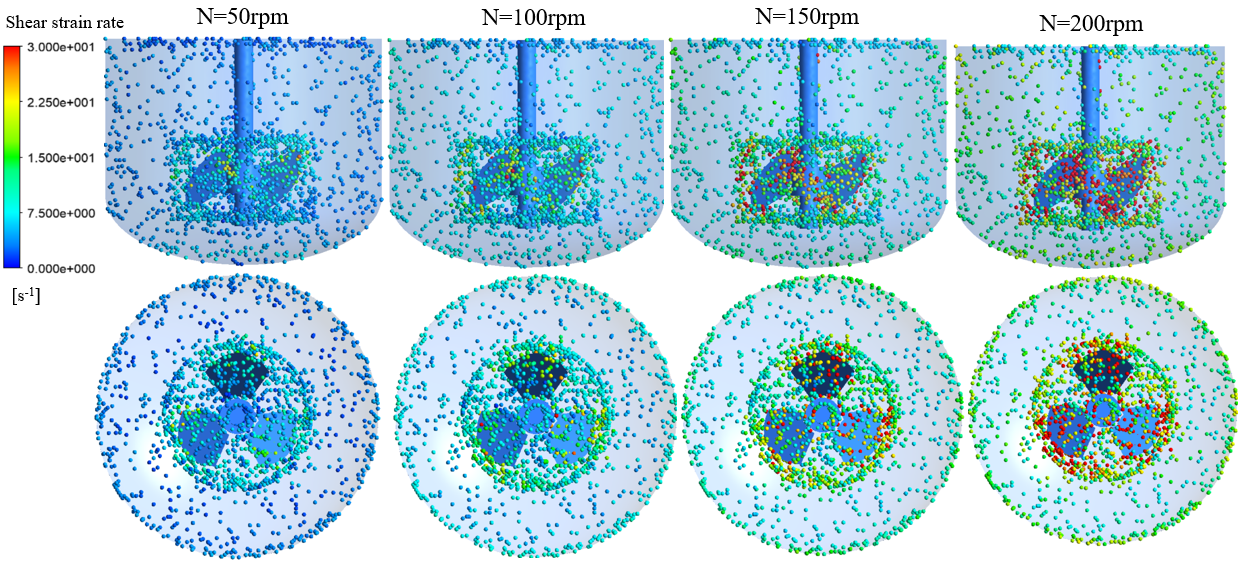


**Fig. S3** Viable cell density (*X*), viability and aggregated cells ratio (A) of BHK-21 cells at different agitation speeds (■: 50 rpm, ●: 100 rpm, ♦: 150 rpm, ▲: 200 rpm) in 5L-EE-0.45 bioreactors (a), 5L-EE-0.5 bioreactors(b), and 5L-PBT-0.5 bioreactors(c).

**Table S1** Fitting models of liquid velocity with agitation speed for different bioreactors.

| Bioreactor types | Fitting Model ^a^ |
| --- | --- |
| 5L-EE-0.45 | $\begin{matrix} U_{imp}=0.1320N-----------R^{2}\text{=}0.9943 \\ U_{\tan}=0.1180N-----------R^{2}\text{=}0.9911 \\ U_{ave}=0.1094N-----------R^{2}\text{=}0.9902 \end{matrix}$ |
| 5L-EE-0.5 | $\begin{matrix} U_{imp}=0.1574N-----------R^{2}\text{=}0.9974 \\ U_{\tan}=0.1336N-----------R^{2}\text{=}0.9933 \\ U_{ave}=0.1365N-----------R^{2}\text{=}0.9942 \end{matrix}$ |
| 5L-PBT-0.5 | $\begin{matrix} U_{imp}=0.1173N-----------R^{2}\text{=}0.9918 \\ U_{\tan}=0.1024N-----------R^{2}\text{=}0.9936 \\ U_{ave}=0.1045N-----------R^{2}\text{=}0.9938 \end{matrix}$ |
| 42L-EE-0.37 | $\begin{matrix} U_{imp}=0.2928N-----------R^{2}\text{=}0.9857 \\ U_{\tan}=0.2337N-----------R^{2}\text{=}0.9995 \\ U_{ave}=0.2375N-----------R^{2}\text{=}0.9995 \end{matrix}$ |
| 350L-EE-0.5 | $\begin{matrix} U_{imp}=0.6017N-----------R^{2}\text{=}0.9949 \\ U_{\tan}=0.5387N-----------R^{2}\text{=}0.9985 \\ U_{ave}=0.5512N-----------R^{2}\text{=}0.9982 \end{matrix}$ |
| 1000L-EE-0.5 | $\begin{matrix} U_{imp}=0.8646N-----------R^{2}\text{=}0.9988 \\ U_{\tan}=0.7658N-----------R^{2}\text{=}0.9954 \\ U_{ave}=0.7873N-----------R^{2}\text{=}0.9970 \end{matrix}$ |

^a^ In the model, N represents the agitation speed with the unit rps (revolutions per second).

**Fig.S2** Fitting models of energy dissipation with agitation speed for different bioreactors.

| Bioreactor types | Fitting Model ^a^ |
| --- | --- |
| 5L-EE-0.45 | $\begin{matrix} \varepsilon_{imp}=0.0021N^{2.395}-----------R^{2}\text{=}0.9777 \\ \varepsilon_{\tan}=0.0005N^{2.4656}-----------R^{2}\text{=}0.9978 \\ \varepsilon_{ave}=0.0007N^{2.4414}-----------R^{2}\text{=}0.9939 \end{matrix}$ |
| 5L-EE-0.5 | $\begin{matrix} \varepsilon_{imp}=0.00191N^{2.8386}-----------R^{2}\text{=}0.9955 \\ \varepsilon_{\tan}=0.0007N^{2.7051}------------R^{2}\text{=}0.9997 \\ \varepsilon_{ave}=0.0008N^{2.7514}------------R^{2}\text{=}0.9988 \end{matrix}$ |
| 5L-PBT-0.5 | $\begin{matrix} \varepsilon_{imp}=0.0017N^{2.2591}-----------R^{2}\text{=}0.9796 \\ \varepsilon_{\tan}=0.0004N^{2.4902}-----------R^{2}\text{=}0.9974 \\ \varepsilon_{ave}=0.0006N^{2.3992}-----------R^{2}\text{=}0.9931 \end{matrix}$ |
| 42L-EE-0.37 | $\begin{matrix} \varepsilon_{imp}=0.0075N^{1.8994}-----------R^{2}\text{=}0.9983 \\ \varepsilon_{\tan}=0.0001N^{2.103}-----------R^{2}\text{=}0.9984 \\ \varepsilon_{ave}=0.0013N^{2.0717}-----------R^{2}\text{=}0.9992 \end{matrix}$ |
| 350L-EE-0.5 | $\begin{matrix} \varepsilon_{imp}=0.0189N^{2.6229}-----------R^{2}\text{=}0.9921 \\ \varepsilon_{\tan}=0.0015N^{2.5155}-----------R^{2}\text{=}0.9957 \\ \varepsilon_{ave}=0.0017N^{2.5478}------------R^{2}\text{=}0.9965 \end{matrix}$ |
| 1000L-EE-0.5 | $\begin{matrix} \varepsilon_{imp}=0.0329N^{2.4832}-----------R^{2}\text{=}0.9993 \\ \varepsilon_{\tan}=0.0029N^{2.1238}-----------R^{2}\text{=}0.9828 \\ \varepsilon_{ave}=0.0031N^{2.2302}-----------R^{2}\text{=}0.9943 \end{matrix}$ |

^a^ In the model, N represents the agitation speed with the unit rps (revolutions per second).

**Fig.S3** Fitting models of shear rate with agitation speed for different bioreactors.

| Bioreactor types | Fitting Model ^a^ |
| --- | --- |
| 5L-EE-0.45 | $\begin{matrix} \gamma_{imp}=6.9705N-----------R^{2}\text{=}0.9788 \\ \gamma_{\tan}=3.1907N-----------R^{2}\text{=}0.9981 \\ \gamma_{ave}=3.5858N-----------R^{2}\text{=}0.9965 \end{matrix}$ |
| 5L-EE-0.5 | $\begin{matrix} \gamma_{imp}=7.9704N-----------R^{2}\text{=}0.9785 \\ \gamma_{\tan}=3.914N------------R^{2}\text{=}0.9984 \\ \gamma_{ave}=4.4824N-----------R^{2}\text{=}0.9957 \end{matrix}$ |
| 5L-PBT-0.5 | $\begin{matrix} \gamma_{imp}=5.7024N-----------R^{2}\text{=}0.9769 \\ \gamma_{\tan}=3.1371N-----------R^{2}\text{=}0.9992 \\ \gamma_{ave}=3.5001N-----------R^{2}\text{=}0.9980 \end{matrix}$ |
| 42L-EE-0.37 | $\begin{matrix} \gamma_{imp}=7.6862N-----------R^{2}\text{=}0.9917 \\ \gamma_{\tan}=3.665N------------R^{2}\text{=}0.9800 \\ \gamma_{ave}=3.9267N-----------R^{2}\text{=}0.9845 \end{matrix}$ |
| 350L-EE-0.5 | $\begin{matrix} \gamma_{imp}=8.7809N-----------R^{2}\text{=}0.9942 \\ \gamma_{\tan}=4.9368N-----------R^{2}\text{=}0.9914 \\ \gamma_{ave}=5.6959N-----------R^{2}\text{=}0.9925 \end{matrix}$ |
| 1000L-EE-0.5 | $\begin{matrix} \gamma_{imp}=11.452N-----------R^{2}\text{=}0.9944 \\ \gamma_{\tan}=4.9371N-----------R^{2}\text{=}0.9954 \\ \gamma_{ave}=6.2274N-----------R^{2}\text{=}0.9963 \end{matrix}$ |

^a^ In the model, N represents the agitation speed with the unit rps (revolutions per second).

**Fig.S4** Fitting models of *EDCF* and circulation time with rotation speed for different bioreactors

| Bioreactor types | Fitting Model ^a^ |
| --- | --- |
| 5L-EE-0.45 | $\begin{matrix} EDCF=5.2871N^{3.881}-----------R^{2}\text{=}0.9975 \\ t_{c}=3.7567N^{-0.866}-------------R^{2}\text{=}0.9998 \end{matrix}$ |
| 5L-EE-0.5 | $\begin{matrix} EDCF=7.6854N^{4.2291}-----------R^{2}\text{=}0.9999 \\ t_{c}=2.4524N^{-0.928}--------------R^{2}\text{=}0.9993 \end{matrix}$ |
| 5L-PBT-0.5 | $\begin{matrix} EDCF=4.3045N^{4.04}-----------R^{2}\text{=}0.9999 \\ t_{c}=3.3541N^{-0.951}-------------R^{2}\text{=}0.9917 \end{matrix}$ |
| 42L-EE-0.37 | $\begin{matrix} EDCF=15.23N^{3.3419}-----------R^{2}\text{=}0.9992 \\ t_{c}=3.4751N^{-0.701}--------------R^{2}\text{=}0.9923 \end{matrix}$ |
| 350L-EE-0.5 | $\begin{matrix} EDCF=21.57N^{3.249}-----------R^{2}\text{=}0.9967 \\ t_{c}=31.748N^{-0.863}-------------R^{2}\text{=}0.9962 \end{matrix}$ |
| 1000L-EE-0.5 | $\begin{matrix} EDCF=35.12N^{3.6675}-----------R^{2}\text{=}0.9999 \\ t_{c}=1.7041N^{-0.976}--------------R^{2}\text{=}0.9966 \end{matrix}$ |

^a^ In the model, N represents the agitation speed with the unit rps (revolutions per second).

**Fig.S5** Fitting models of *K_L_a* with agitation speed for different bioreactors.

| Bioreactor types | Fitting Model ^a^ |
| --- | --- |
| 5L-EE-0.45 | $K_{L}a=2.3095N^{0.45}------------R^{2}\text{=}0.9770$ |
| 5L-EE-0.5 | $K_{L}a=2.7388N^{0.345}------------R^{2}\text{=}0.9470$ |
| 5L-PBT-0.5 | $K_{L}a=2.4208N^{0.428}------------R^{2}\text{=}0.9680$ |
| 42L-EE-0.37 | $K_{L}a=6.6476N^{0.1316}------------R^{2}\text{=}0.9587$ |
| 350L-EE-0.5 | $K_{L}a=10.072N^{0.1731}------------R^{2}\text{=}0.9754$ |
| 1000L-EE-0.5 | $K_{L}a=12.342N^{0.2168}------------R^{2}\text{=}0.9532$ |

^a^ In the model, N represents the agitation speed with the unit rps (revolutions per second).
